# Contact Tracing Different Age Groups During the COVID-19 Pandemic: Retrospective Study From South-West Germany

**DOI:** 10.2196/54578

**Published:** 2024-10-29

**Authors:** Christopher Michael Dyer, Alexandra-Teodora Negoescu, Matthias Borchert, Christoph Harter, Anne Kühn, Peter Dambach, Michael Marx

**Affiliations:** 1 Rhein-Neckar District and Heidelberg City Public Health Authority Heidelberg Germany; 2 Medical Faculty Heidelberg University Heidelberg Germany; 3 Heidelberg Institute of Global Heath University Hospital Heidelberg University Heidelberg Heidelberg Germany

**Keywords:** COVID-19, SARS-CoV-2, pandemic, quarantine, contact tracing, contact tracing effectiveness, demographics, mortality, case fatality, public health surveillance, public health systems

## Abstract

**Background:**

Contact tracing was implemented in many countries during the COVID-19 pandemic to prevent disease spread, reduce mortality, and avoid overburdening health care systems. In several countries, including Germany, new systems were needed to trace potentially infected individuals.

**Objective:**

Using data collected in the Rhine-Neckar and Heidelberg (RNK/HD) districts in southwest Germany (population: 706,974), this study examines the overall effectiveness and efficiency of contact tracing in different age groups and stages of the pandemic.

**Methods:**

From January 27, 2020, to April 30, 2022, the RNK/HD Health Authority collected data on COVID-19 infections, quarantines, and deaths. Data on infection, quarantine, and death was grouped by age (young: 0-19 years; adult: 20-65 years; and senior citizens: >65 years) and pandemic phase (infectious wave plus subsequent lull periods) and analyzed for proportion, risk, and relative risk (RR). The overall effectiveness and efficiency of contact tracing were determined by calculating quarantine sensitivity (proportion of the infected population captured in quarantine), positive predictive value (PPV; proportion of the quarantined population that was infected), and the weighted Fβ-score (combined predictive performance).

**Results:**

Of 706,974 persons living in RNK/HD during the study period, 192,175 (27.2%) tested positive for SARS-CoV-2, 74,810 (10.4%) were quarantined, and 932 (0.132%) died following infection. Compared with adults, the RR of infection was lower among senior citizens (0.401, 95% CI 0.395-0.407) and while initially lower for young people, was ultimately higher for young people across all 5 phases (first-phase RR 0.502, 95% CI 0.438-0.575; all phases RR 1.35, 95% CI 1.34-1.36). Of 932 COVID-19–associated deaths during the study period, 852 were senior citizens (91.4%), with no deaths reported among young people. Relative to adults, senior citizens had the lowest risk of quarantine (RR 0.436, 95% CI 0.424-0.448), while young people had the highest RR (2.94, 95% CI 2.90-2.98). The predictive performance of contact tracing was highest during the second and third phases of the pandemic (Fβ-score=0.272 and 0.338, respectively). In the second phase of the pandemic, 5810 of 16,814 COVID-19 infections were captured within a total quarantine population of 39,687 (sensitivity 34.6%; PPV 14.6%). In the third phase of the pandemic, 3492 of 8803 infections were captured within a total quarantine population of 16,462 (sensitivity 39.7%; PPV 21.2%).

**Conclusions:**

The use of quarantine aligned with increasing risks of COVID-19 infection and death. High levels of quarantine sensitivity before the introduction of the vaccine show how contact tracing systems became increasingly effective at capturing and quarantining the infected population. High levels of PPV and Fβ-scores indicate, moreover, that contact tracing became more efficient at identifying infected individuals. Additional analysis of transmission pathways is needed to evaluate the application of quarantine in relation to infection and death risks within specific age groups.

## Introduction

### Background

SARS-CoV-2 was first identified as the causative agent of the respiratory disease COVID-19 in Wuhan, Hubei Province, China, in December 2019 [1,2]. As of April 30, 2022, over half a billion cases of COVID-19 and more than 6 million COVID-19–related deaths have been reported worldwide [3].

### COVID-19 in Germany

The first COVID-19 case in Germany was identified in Munich on January 27, 2020. By the end of April 2022, Germany, with a population of approximately 83 million, had registered over 24 million COVID-19 cases and more than 135,000 COVID-19–related fatalities. While the disease affected all age groups, nearly 90% of all COVID-19–related deaths in Germany were among individuals aged over 60 years, and over 98% were among those aged over 50 years [4]. According to Germany’s national public health institute, the Robert Koch Institute (RKI), the majority of the initial SARS-CoV-2 infections in Germany occurred in 6 distinct waves, each primarily dominated by 1 of 4 virus variants [5].

During the first 2 waves of the COVID-19 pandemic, which were predominantly driven by the wild-type variant, studies generally reported a higher risk of infection in adults compared with younger individuals [6-8]. Additionally, there was a higher risk of infection in school-aged children (6-14 years) compared with those in childcare and kindergarten (0-5 years) [9]. Subsequent seroprevalence studies, however, have suggested that COVID-19 infections among younger individuals may have been underreported, as children and adolescents were often asymptomatic during a period of limited testing capacity [10,11]. Conversely, reduced transmission rates may have contributed to lower infection rates in younger people during the first 2 waves of the pandemic dominated by the wild-type variant [12-14]. Data from the Corona-KiTa report, published by the RKI and the German Youth Institute (DJI), also indicate that during the first 2 infection waves, the incidence of disease among children and adolescents, particularly those aged 0-5 years, was not only lower but also exhibited a delayed peak compared with older populations. The infection wave for this specific age group rose later and declined earlier than in older age groups [9].

### Contact Tracing and Vaccination as Infection Control Measures

Infection control measures are specific actions designed to stop or reduce the spread of disease, thereby decreasing associated morbidity, mortality, and the burden on health care systems. During the COVID-19 pandemic in Germany, these measures were defined at both the federal and state levels and implemented by district health authorities. In addition to contact tracing and vaccination, which were critical control measures throughout the pandemic, other measures included lockdown periods, travel restrictions, and nightly curfews; temporary closure of schools, businesses, and religious institutions; prohibition of social, cultural, and sporting events; personal hygiene measures (eg, handwashing and sanitization); limitations on social contacts and physical distancing (1.5 m); and case isolation [15].

Most infection control measures were applied broadly across the entire population and often specifically targeted young people due to concerns about severe outcomes [16-21], including “long COVID” [22] and the risk of transmission to high-risk groups based on social contact data [23-26]. While these measures were generally effective, they were associated with increased levels of stress [27], language difficulties, weight gain [28], anxiety [29], stigmatization [30], depression [31], reduced sleep quality [9,28,32], and lower intelligence test scores [33]. Particularly among children and young people, the proportion of individuals with mental health problems dramatically increased during the pandemic [31,34,35]. Young people also experienced decreased social and physical activity [32], coupled with increased media consumption [36], as well as higher rates of overweight and obesity [37-39]. Further studies reported declines in language development and fine motor skills in children [28]. Among adults and senior citizens, studies have highlighted increases in economic difficulties, such as loss of income or savings, and reduced access to health and other essential services [32]. For parents of young children, limited access to childcare facilities was identified as a significant factor contributing to increased stress and anxiety [9]. Reports indicated rises in all forms of domestic violence, including sexual violence [40,41], amid concerns that prolonged confinement could be a risk factor for heightened family conflicts and child mistreatment [15,42]. German authorities closely monitored the negative impacts of infection control measures, particularly on children and adolescents’ education, fitness, social adjustment, and mental health [15,43].

Contact tracing and quarantine are specific infection control measures that involve identifying and isolating individuals who have been in close contact with someone diagnosed with a transmissible disease. This differs from isolating a known or confirmed case. Unlike many countries, Germany’s well-defined infection surveillance processes [44,45] were shown to have detected and reported close to the true number of COVID-19 cases during the pandemic [46,47]. This was achieved through a broad range of measures, including disease education campaigns and daily status updates to raise awareness of periods of heightened infectivity; automated contact tracing solutions, such as the Corona Warn App (SAP/Deutsche Telekom), which alerted users to their proximity to a known infection; event check-in systems, including mobile apps (eg, Luca app, culture4life GmbH), to identify potential disease transmission; and subsidized home testing along with widespread free antigen and PCR testing sites that facilitated easy access to infection status information. While antigen test results were not used for reporting or contact tracing during the pandemic in Germany, a positive result from an antigen test generally prompted subsequent PCR testing. This process increased the detection of confirmed cases within the community and among quarantined populations.

Laboratories were required, under the Infectious Disease Protection Act (Infektionsschutzgesetz, §§ 7 to 9 IfSG [48]), to report each confirmed case to local health authorities. Specially trained contact tracing staff then attempted to contact and conduct telephone interviews with all COVID-19 cases to identify close contacts, also known as contact persons. They also informed individuals of their legal requirement to isolate and collected additional medical information. If the health authority was unable to establish contact with a known case, the local regulatory authority was notified, and an officer was dispatched to make contact. Ultimately, the Rhein-Neckar-Kreis/Heidelberg (RNK/HD) Health Authority estimated that during the period of active contact tracing, it successfully established contact with 85% of COVID-19 cases, with an additional 5% reached through the local regulatory authority. Additional contact tracing measures were implemented by specialist teams to protect senior citizens in care facilities and young people in kindergartens and schools. Once identified, close contacts were reached by contact tracing staff and informed of their legal obligation to quarantine in their residence, ideally in a separate room away from other coinhabitants, for a period ranging from 5 to 14 days. The definition of close contact and the duration of quarantine were specified and updated throughout the pandemic by state governments in accordance with national RKI guidelines [49]. To ensure compliance, officers from the local regulatory authority investigated reports of potential breaches and conducted spot inspections of disease case isolations and quarantined individuals. Although rarely issued in RNK/HD, regulatory officers had the authority to impose fines of up to €25,000 (~US $27731) or impose prison sentences of up to 5 years in extreme cases where there was a significant risk of harm to others (§ 74, 75 IfSG [48]).

Vaccines to prevent the spread and minimize the impact of COVID-19 were approved for emergency use in Germany on December 21, 2020, following recommendations from the European Medicines Agency (EMA; Table 1). The RNK/HD Health Authority administered the first doses in the district on December 27, 2020 [50].

**Figure 1:**
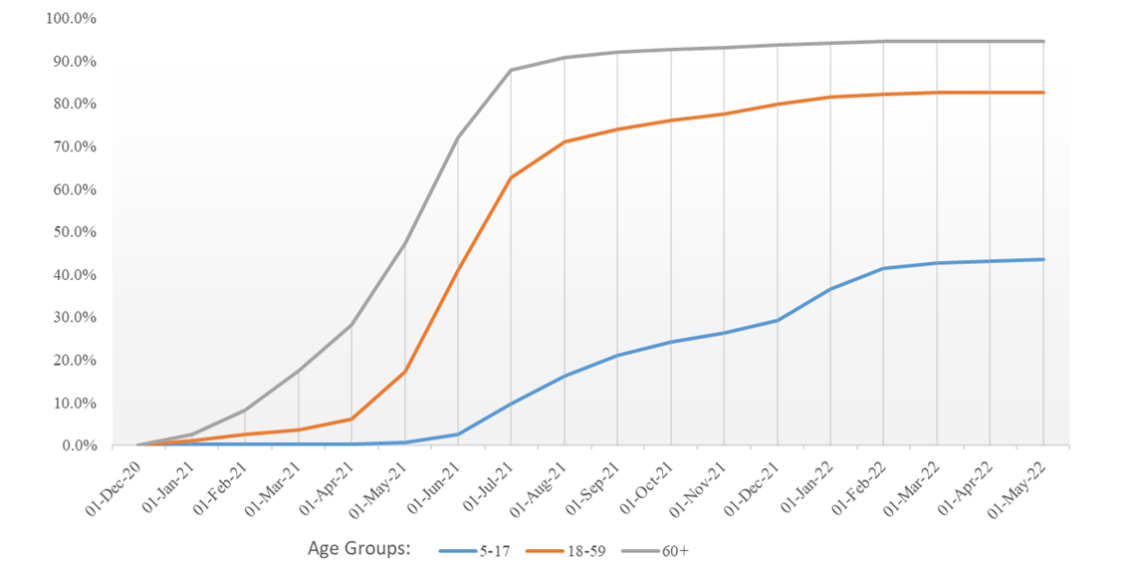
COVID-19 vaccination coverage in RNK/HD from January 2021 to May 2022 [52]. HD: Heidelberg; RNK: Rhine-Neckar.

As part of a national strategy to prioritize limited vaccine supply for senior citizens and high-risk individuals [50], the RNK/HD Health Authority initially administered vaccinations through 3 immunization centers in the RNK/HD district and mobile teams focused on aged care facilities and other vulnerable groups. Vaccines became more broadly available through medical practices starting April 7, 2021, and were later offered in pharmacies beginning February 8, 2022. Prioritized distribution of the vaccine ended, and young people over the age of 12 years were granted access starting June 7, 2021, following approval by the EMA [51], despite a lack of recommendation from the National Vaccine Advisory Authority (Standing Committee on Vaccination [STIKO]) [52]. While vaccination was mandatory for individuals working in medical or aged care facilities, the general population was encouraged to get vaccinated through social responsibility education programs and incentive schemes (Figure 1; also see [53]).

**Figure 2:**
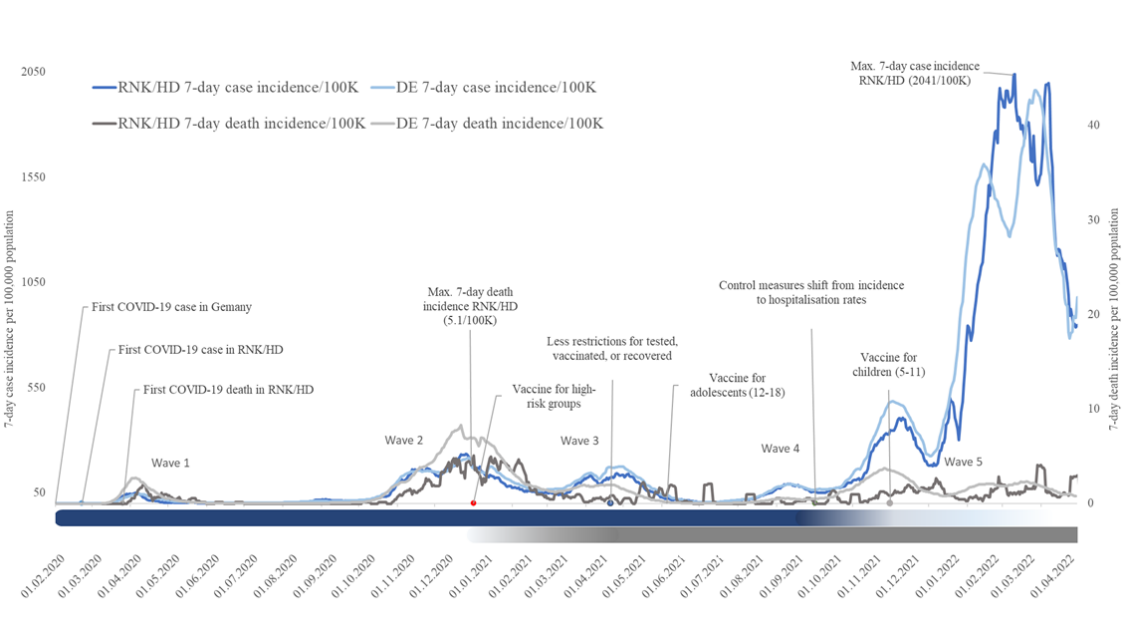
Timeline of initial COVID-19 disease incidence (morbidity) and death (mortality), with critical events including period of contact tracing and the introduction of the vaccine in RNK/HD, Germany. DE: Germany; HD: Heidelberg; RNK: Rhein-Neckar-Kreis.

Contact tracing efforts and quarantine measures in RNK/HD were scaled back toward the end of 2021, following the widespread adoption of the COVID-19 vaccine. By this time, many other infection control measures that limited social contact had been lifted for individuals who could demonstrate a negative test result, proof of vaccination, or evidence of a recent infection within the last 90 days. As case fatalities remained relatively low following the Omicron-dominated fifth wave and hospital and intensive care unit capacities were no longer critical in 2022, most institutions and businesses gradually resumed normal operations while adhering to remaining infection control measures. At the end of March 2022, the RNK/HD Health Authority ceased active contact tracing, and on November 15, 2022, the state government of Baden-Württemberg lifted the legal requirement for quarantine (Figure 2).

**Table 1:** COVID-19 vaccine approvals.

Registered name	Manufacturer	Technology	Approval date
Comirnaty	BioNTech/Pfizer	mRNA^a^	December 21, 2020
Spikevax	Moderna	mRNA	January 6, 2021
Vaxzevria	Oxford/AstraZeneca	Adenovirus vector	January 29, 2021
Ad26.COV2.S	Janssen (Johnson & Johnson)	Adenovirus vector	March 11, 2021
Nuvaxovid	Novavax	NVX-CoV2373	December 20, 2021

^a^mRNA: messenger RNA.

### Measuring Contact Tracing Effectiveness and Efficiency

A Cochrane rapid review on quarantine during the COVID-19 pandemic concluded that, while broadly accepted as effective, there is a notable lack of agreed-upon metrics and data to demonstrate its effectiveness [54]. This finding was supported by a subsequent systematic review of observational and modeling studies of COVID-19, which concluded that the spread of the disease could be stopped if at least 80% of cases were captured in quarantine, or slowed if the capture rate was below 80% [55].

**Figure 3:**
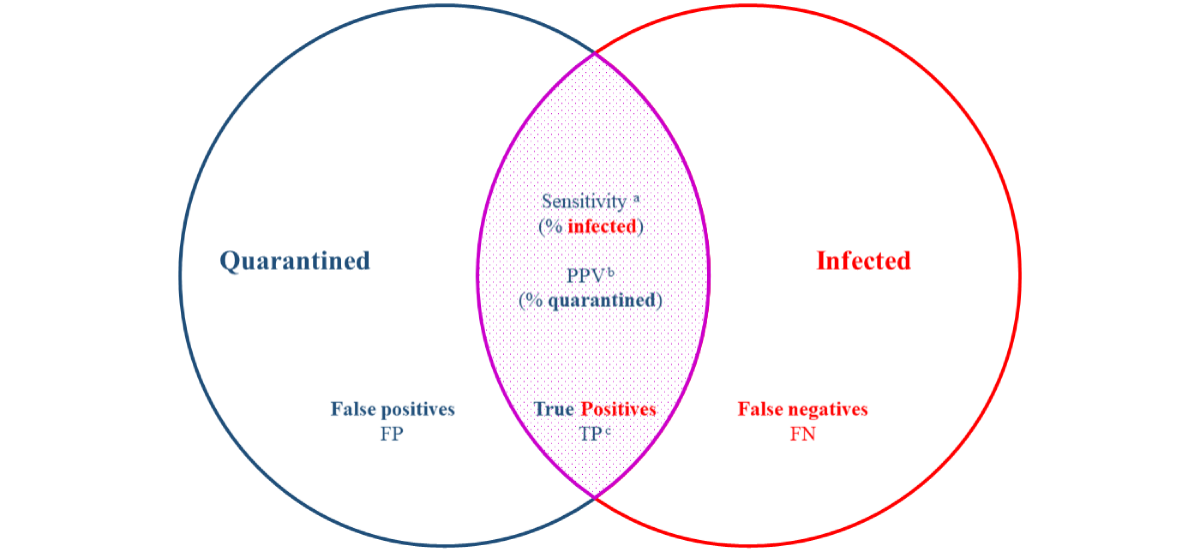
Quarantine sensitivity, PPV, and predictive performance. ^a^Sensitivity = cases captured in quarantine/total number of infected cases. ^b^PPV = cases captured in quarantine/total number of people quarantined. ^c^Predictive performance (<i>F</i>_β_-score) = ([1+β^2^]TP)/([1+β2]TP+β2FN+FP). FN: false negative; FP: false positive; PPV: positive predictive value; TP: true positive.

This paper examines the use of quarantine as an infection control measure in RNK/HD from the initial disease outbreak until the introduction of the vaccine. To assess overall effectiveness in a real-life setting, we propose using “quarantine sensitivity,” defined as the proportion of the infected population captured in quarantine (Figure 3). To assess overall efficiency, we propose using the positive predictive value (PPV), defined as the proportion of the quarantined population that tested positive while in quarantine. To evaluate predictive performance as a combined measure of sensitivity and PPV, we propose using an *F*_β_-score, with an arbitrary weight that values sensitivity more than PPV. Ideally, all infected individuals would be quarantined (sensitivity 100%) and only infected individuals would be quarantined (PPV 100%), resulting in perfect predictive performance (*F*_β_-score=1). In practice, quarantine can be considered overall effective if it captures a sufficient proportion of the infected population to limit or reduce disease spread. It is deemed efficient if the number of healthy individuals placed in quarantine is kept to tolerable levels.

### Study Objectives

The study focuses on contact tracing and the use of quarantine within 3 distinct age groups during the initial 5 phases of the COVID-19 pandemic. In particular, did the use of quarantine reflect variations in COVID-19 infection and mortality between the different age groups and at different time points? Was contact tracing effective in identifying and capturing potentially infected individuals in quarantine? How efficient was contact tracing in capturing only infected individuals in quarantine? Were there differences between age groups and across the different phases of the pandemic?

## Methods

### Data Collection and Storage

All known COVID-19 cases, including the first cases reported in RNK on February 27, 2020, and in HD on February 28, 2020, were reported to district health authorities within 24 hours. The RNK/HD District Health Authority then transmitted case data to the RKI while managing the infectious disease control process. Initial containment measures were managed largely through ad hoc systems until a dedicated COVID-19 monitoring and containment system, including an operational database, was developed and launched on March 8, 2020. A database for research purposes was subsequently created from the comprehensive outputs (CSV files) of the operational database, which were archived daily until November 2022.

Until August 2021 (the end of the third phase), the RNK/HD Health Authority contacted each confirmed case via telephone and regular email to inform them of their legal obligation to isolate, collect medical information, and identify potentially infected contacts. RNK/HD contact tracing staff received specialized training and followed a standardized procedure to collect and document case details for national reporting, contact tracing, and other operational purposes.

This study includes data from the first documented infection on February 27, 2020, until the cessation of contact tracing in the region on April 30, 2022. Medical records collected by the RNK/HD Health Authority without informed consent were anonymized, aggregated, and analyzed in accordance with the EU General Data Protection Regulation (GDPR 2016/679), specifically recitals 1, 4, 26, and 159 [56], and the Treaty on the Functioning of the European Union, Title XIX, Research and Technological Development Space, Article 179 [57].

### Ethics Approval

The use of patient data records by health authority staff without informed consent was approved for this study by the University of Heidelberg Medical Faculty Ethics Committee on September 30, 2022 (reference number S-488/2022).

Additional national COVID-19 data and statistics, including vaccination levels, were accessed from the RKI national database on February 1, 2023, and other publicly released sources [4]. Population data for RNK/HD, valid as of December 31, 2020, were obtained from the Baden-Württemberg Office for Statistics web page, accessed on October 6, 2022 [58].

### Documentation and Outcomes

For reporting purposes, a confirmed COVID-19 case was defined as an individual with a positive SARS-CoV-2 reverse transcription polymerase chain reaction (RT-PCR) test, regardless of symptoms [59]. Individuals who were in close contact with a confirmed case and were legally required to undergo quarantine were contacted separately and documented as noncases within the COVID-19 monitoring and containment system. A death was documented as COVID-19 related if the person tested positive for COVID-19 via RT-PCR either before or immediately after death, and if medical professionals assessed that COVID-19 contributed to or caused the death.

In the research database, a confirmed case was documented with the date of disease onset as the testing date, or with the date of the first positive PCR result if no actual test date was recorded in the operational database. Contact persons were documented as nonconfirmed cases, with a quarantine start date based on when they were first entered into the operational database, and an end date determined by the median duration of quarantine for each specific day during the study period. Infected contact persons were identified if the date of disease onset occurred during the quarantine period. Noninfected contact persons were those who did not become infected during the quarantine period, although they may have been infected at another time. To avoid double counting across study phases, records of infected contact persons who were quarantined in one phase but tested positive or died in another phase were included only in the phase during which they were quarantined. Similarly, for nonquarantined persons, if death occurred in a subsequent study phase after the infection, the record was included only once, in the phase of the infection.

### Statistical Analysis

This publication presents data collected by the RNK/HD Health Authority, the sixth-largest district health authority in Germany, which serves a combined population of 706,974 registered residents as of December 31, 2020 [58].

Data were included from the first case in the RNK/HD area, recorded on February 27, 2020, until April 30, 2022, by which time many infection control measures, including contact tracing, had been relaxed. The study timeframe was divided into 5 phases (Table 2), with each phase beginning at the start of a calendar week in which incidence rates increased due to a new virus variant or following a summer lull in infection levels, according to RKI retrospective classification [5]. Lulls are included at the end of a phase because quarantine measures, infections, deaths, and the emergence of new virus variants were more frequently initiated during infection waves that extended into lulls, rather than during lulls that extended into waves.

Data were further categorized into 3 age groups: (1) young people, which included predominantly those attending kindergarten and school (aged 0-19 years, n=130,387); (2) adults, which included predominantly higher education students, parents, and those engaged in the workforce (retirement age in Germany: 65 years, aged 20-65 years, n=437,581); and (3) senior citizens, which included predominantly retired persons, including those in aged care facilities and individuals at high risk of hospitalization and fatal outcomes from COVID-19 [60] (aged ≥66 years, n=139,006).

The risks of COVID-19–related quarantine, infection, and death were calculated as follows: the number of individuals who were quarantined, infected, or who died with or of COVID-19 during the specified period was divided by the relevant subpopulation as of December 31, 2020 [58]. All risk calculations consider only single instances during the specified time and exclude additional instances where an individual may have been quarantined or infected multiple times. Reinfections and/or multiple quarantine events were counted as separate events. The adult age group, which constitutes 61.89% (437,581/706,974) of the population, was used as the reference group to calculate the relative risk (RR). The corresponding 95% CI was calculated using a Wald test with bivariable logistic regression, assuming a normal distribution.

Using a contingency table to evaluate binary classifiers, the sensitivity of contact tracing or quarantine decisions was calculated as the percentage of COVID-19 cases captured in quarantine (true positives [TPs]/(TPs + false negatives [FNs]). The PPV of quarantine was calculated as the percentage of persons quarantined who tested positive for COVID-19 during the quarantine period (TP/(TP + false positive [FP]). As a combined measure of predictive performance, *F*_β_-scores were weighted toward sensitivity rather than PPV (β=2), and calculated as the weighted product of PPV and sensitivity divided by weighted PPV plus sensitivity, which can be simplified as follows: ([1+β^2^]TP)/([1+β2]TP+β2FN+FP). It was not possible to calculate test accuracy because the number of true negatives (contacts, but not close contracts, of infected persons) was not documented.

Records without birth dates or with implausible birth dates were excluded from age-related analyses, including 988 of 78,641 quarantine records (1.26%) and 34 of 198,148 case records (0.02%).

To validate the results, supplementary risk analyses for infection, quarantine, or death were conducted by grouping data based on gender and location (HD city vs surrounding RNK district). No statistically significant differences (within 95% CI) were expected between males and females, but some variations were anticipated between RNK and HD due to demographic differences.

In this report, comparable output values ranging from 0.001 to 999 were rounded to 3 significant digits (eg, 12.3, 1.23, 0.123, 0.012, and 0.001). A decimal 0 was added to natural numbers to indicate that rounding had occurred (eg, 12.0, 1.20).

Data processing and analysis for this report were conducted using MS-SQL (Microsoft Corporation) and Python version 3.12 (Python Foundation) [61]. All statistical assessments were performed using pandas version 2.1.1 [62], statsmodels version 0.14.0 [63], scipy version 1.11.3 [64], and matplotlib version 3.8.4, with matplotlib-venn version 0.11.10 [65] for Venn diagrams. Additional graphs and tables were prepared using MS Excel 2016 (Microsoft Corporation) [66].

**Table 2:** The initial 5 phases of the COVID-19 pandemic in Germany [5].

Study phase (period) and RKI^a^ phase description			Dominant viral strain
**1: February 27, 2020 to September 27, 2020**			
	Sporadic cases	Wild type	
	First wave	Wild type	
	Summer lull	Wild type	
**2: September 28, 2020 to February 28, 2021**			
	Second wave	Wild type	
**3: March 1, 2021 to August 1, 2021**			
	Third wave	Alpha	
	Summer lull	Alpha	
**4: August 2, 2021 to December 26, 2021**			
	Fourth wave	Delta	
**5: December 27, 2021 to April 30, 2022**			
	Sixth wave	Omicron	

^a^RKI: Robert Koch Institute.

## Results

### Age Group Proportion of Infection, Quarantine, and Case Fatalities

**Figure 4:**
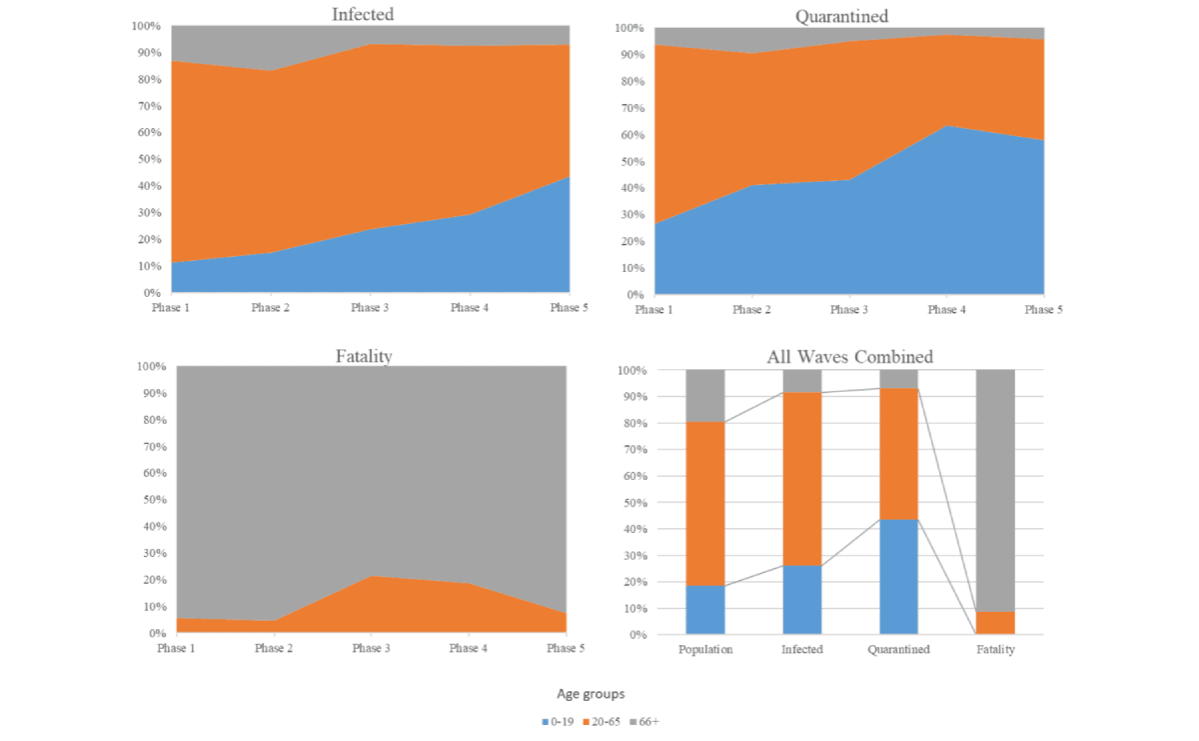
Proportion of COVID-19-related infections, quarantine, and death cases, by age group and pandemic phase (stacked areas) from January 27, 2020, to April 30, 2022.

Within the total population of 706,974 people, young people aged 0-19 years account for 18.41% of the population (n=130,137) and were underrepresented among cases during the first 2 phases of the pandemic, comprising 11.23% (phase 1, 234/2083) and 15.02% (phase 2, 2513/16,730) of confirmed cases (Figure 4; Supplementary Table S2 in Multimedia Appendix 1). However, over the course of the 5 pandemic phases, young people became overrepresented as cases, eventually accounting for 26.31% (50,560/192,141) of COVID-19 infections. Adults aged 20-65 years, who account for 61.9% (n=437,581) of the total population, were slightly overrepresented in the infected population, accounting for 65.48% (125,822/192,141) of cases. This overrepresentation was particularly pronounced in the first phase of the pandemic, where they made up 75.5% (1574/2083) of COVID-19 infections. By contrast, senior citizens aged over 65 years, who account for 19.7% (n=139,006) of the total population were underrepresented in the infected population, comprising 8.46% (16,263/192,141) of cases.

Young people, as 18.4% of the local population, were overrepresented in the quarantined population, making up 43.47% (32,092/73,822) of those quarantined (Figure 4; Supplementary Table S2 in Multimedia Appendix 1). This overrepresentation increased from 26.56% (2046/7702) in the first phase to a peak of 63.43% (5178/8163) in the fourth phase. By contrast, both adults and senior citizens were underrepresented in the quarantined population. Adults, 61.9% of the population, accounted for 49.46% (36,510/73,822) of those quarantined and senior citizens, 19.7% of the population, accounted for 7.07% (5220/73,822) of quarantines. This underrepresentation was particularly pronounced in the fourth phase, where adults made up 34.13% (2786/8163) of those quarantined and senior citizens just 2.44% (199/8163) of those quarantined.

The vast majority of COVID-19–associated deaths documented by the RNK/HD Health Authority occurred within the population of senior citizens, aged over 65 years, accounting for 91.4% (852/932) of all COVID-19 deaths across all study phases (Figure 4; Supplementary Table S2 in Multimedia Appendix 1). The remaining deaths occurred in adults aged 20-65 years (80/932, 8.6%), with no deaths reported among younger people aged 0-19 years. During the third and fourth phases, adults represented relatively higher proportions of the COVID-19 deaths: 22% (14/65) and 19% (26/140), respectively, compared with the first, second, and fifth phases, where they accounted for 5.7% (3/53), 4.7% (22/469), and 7.3% (15/205) of deaths, respectively.

### Risks of Infection, Quarantine, Mortality, and Case Fatality

**Figure 5:**
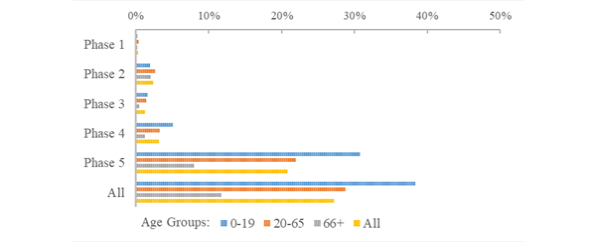
Age group risk of COVID-19 infection during the intial 5 phases of the pandemic.

Within the RNK/HD population of 706,974 people, a total of 198,148 SARS-CoV-2 infections were documented during the study period (Supplementary Table S1 in Multimedia Appendix 1). The lowest risk of infection occurred during the first phase of the pandemic (infections, n=2083, risk 0.295%). In this phase, the risk of COVID-19 infection was 0.181% for young people (infections, n=236), 0.361% for adults (infections, n=1578), and 0.196% for senior citizens people (infections, n=273; Figure 5; Supplementary Table S3 in Multimedia Appendix 1). The risk of infection reached its highest level of 20.8% (infections, n=147,866), which occurred during the omicron-dominated fifth phase. In this phase, 31.0% of young people (n=40,476), 22.0% of adults (n=96,273), and 7.98% of senior citizens (n=11,093) tested positive for COVID-19. During the period of active contact tracing (phases 1-3) in RNK/HD, the highest infection risk for all age groups was observed in the second phase of the pandemic.

For each age group, the lowest risk of infection occurred during the first phase of the pandemic, while the greatest risks were observed in the fifth phase (Figure 5). During the initial 2 phases, young people had a lower risk of infection compared with adults (RR 0.502 in phase 1 and 0.745 in phase 2; Supplementary Table S3 in Multimedia Appendix 1). However, across all phases, young people experienced a higher risk of infection than adults (RR 1.35, 95% CI 1.34-1.36). Senior citizens consistently maintained a significantly lower risk of infection compared with adults throughout all 5 phases (RR 0.401, 95% CI 0.395-0.407).

**Figure 6:**
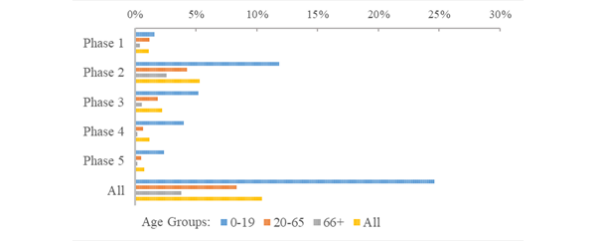
Age group risk of quarantine during the initial 5 phases of the COVID-19 pandemic.

**Figure 7:**
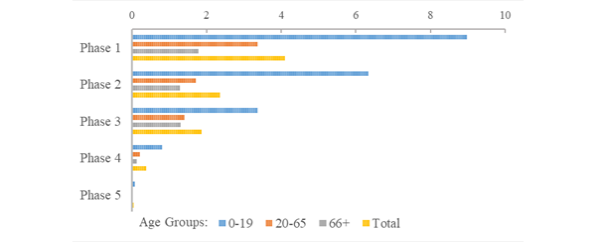
Ratio of quarantine events to COVID-19 cases during the initial 5 phases of the pandemic.

The risk of being placed in quarantine during the first five phases of the pandemic in the RNK/HD area was just over 10% (quarantines, n=78,641; risk 10.4%). The greatest risk of being quarantined occurred in the second phase of the pandemic (quarantines, n=39,687, 5.32%; Figure 6; Supplementary Table S3 in Multimedia Appendix 1). The ratio of quarantine events to confirmed cases was highest in phase 1, decreasing in each successive phase of the pandemic from 4.10 in phase 1 to 0.036 in phase 5; and highest for young people (ranging from 8.97 to 0.077; Figure 7). The population of senior citizens consistently had the lowest ratio of quarantine events to confirmed cases across all phases of the pandemic, ranging from 1.78 in phase 1 to 0.020 in phase 5. Young people had the highest risk of quarantine (quarantines, n=33,761; risk 5.9%) compared with adults (quarantines, n=38,553; risk 8.81%) and senior citizens (quarantines, n=5339; risk 3.84%). Relative to adults, the risk of quarantine for young people was nearly 3 times higher (RR 2.94, 95% CI 2.90-2.98), while the risk of quarantine for senior citizens was less than half (RR 0.436, 95% CI 0.424-0.448).

**Figure 8:**
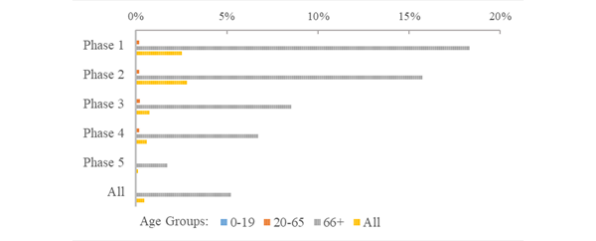
Age group risk of death following COVID-19 infection (case fatality) during the initial 5 phases of the pandemic.

**Figure 9:**
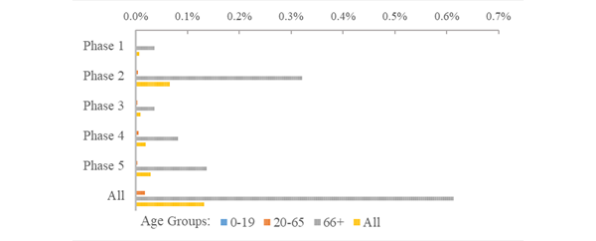
Age group risk of COVID-19–related death (mortality) during the initial 5 phases of the pandemic.

The overall risk of death following infection (case fatality) was 0.485% (deaths, n=932; infections, n=192,141) during the study period, with a notable decline across successive phases of the pandemic, from 2.54% in phase 1 (deaths, n=53; infections, n=2083) to 0.139% in phase 5 (deaths, n=205; infections, n=147,050; Figure 8; Supplementary Table S3 in Multimedia Appendix 1). For the senior citizen population, the case fatality risk dropped significantly, from 18.3% in phase 1 (deaths, n=50; infections, n=273) to 1.72% in phase 5 (deaths, n=190; infections, n=11,037). Within the population of 706,974 people, the population-wide mortality risk during the first five waves of the pandemic was 0.132% (deaths, n=932), with senior citizens experiencing a higher mortality risk at 0.613% (deaths, n=852; senior citizen populations, n=139,006). The mortality risk peaked during the second phase of the pandemic, with 0.066% for all age groups (deaths, n=469) and 0.322% for senior citizens (deaths, n=447; Figure 9). Senior citizens had a significantly higher risk of COVID-19 mortality compared with adults, with the risk being between 11.5 and 64.0 times greater across the study phases. The lowest RR for senior citizens was observed in the third phase of the pandemic, which followed the targeted release of the vaccine to senior citizens and high-risk individuals.

Supplementary analyses aimed at validating the results indicated no significant differences between males (population, n=345,903; infections, n=94,993; risk 27.46%) and females (population, n=362,072; infections, n=102,328; risk 28.26%) in terms of infection risk (RR infection female relative to male 1.03, 95% CI 1.02-1.04), quarantine risk (male quarantines, n=36,499, male risk 10.55%, female quarantines, n=39,724, female risk 10.97%; RR quarantine female relative to male 1.04, 95% CI 1.03-1.06), or death risk (male deaths, n=447, male risk 0.13%; female deaths, n=479, female risk 0.13%; RR death female relative to male 1.03, 95% CI 0.903-1.17; Supplementary Table S4 in Multimedia Appendix 1). Compared with 158,741 residents of HD, those 548,233 persons living in the surrounding RNK district had slightly higher risks of infection (HD infections, n=39,637, HD risk 24.97%; RNK infections, n=157,084, RNK risk 28.65%; RNK RR to HD 1.15, 95% CI 1.14-1.16), quarantine (HD quarantine, n=14,317, HD risk 9.02%; RNK risk 114%; RNK RR to HD 1.26, 95% CI 1.24-1.29), and death (HD deaths 145, HD risk 0.09%; RNK deaths 774, risk 0.14%; RNK RR to HD 1.55, 95% CI 1.30-1.85).

An additional analysis of RNK/HD data confirmed results from the RKI Corona-KiTa study [9], showing that the risk of infection in young children (aged 0-5 years) remained below the risk of infection in adults in each phase of the pandemic until April 30, 2022 (Supplementary Table S5 in Multimedia Appendix 1). Despite having a lower risk of infection than adults and no associated deaths, young children aged 0-5 years had the highest risk of being placed in quarantine (risk 28.5%; RR to adults 3.23, 95% CI 3.18-3.29).

### Quarantine Sensitivity, Positive Predictive Value, and Predictive Performance

**Figure 10:**
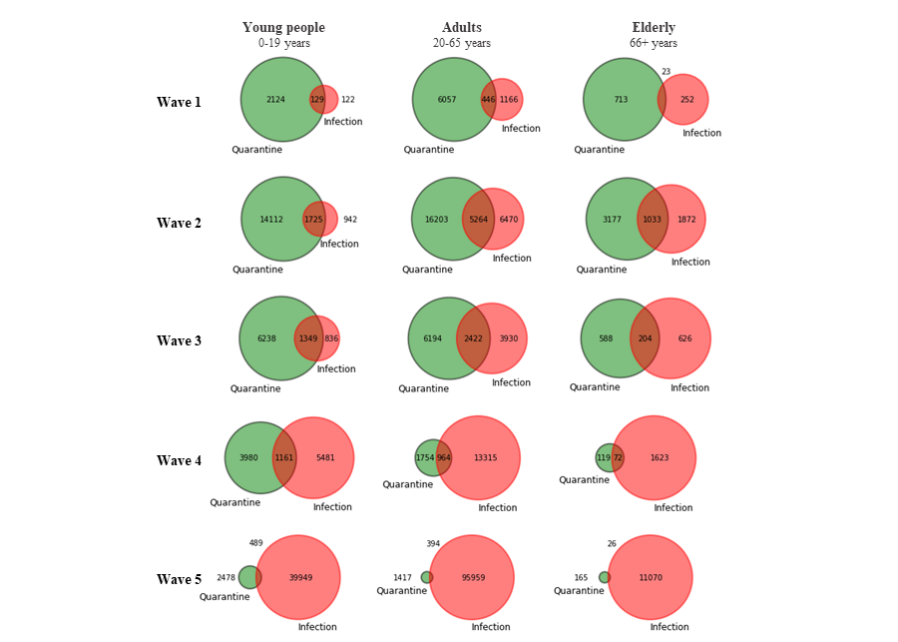
Proportional Venn diagrams of COVID-19 infections captured in quarantine (sensitivity) and within quarantined population (PPV) providing elements for predictive performance (Fβ-score) calculations in age groups and phases of the pandemic. PPV: positive predictive value.

The size of the quarantine population was larger than the size of the infected population during the first 3 phases of the pandemic, with increasing overlap, particularly for young people, between the 2 populations (sensitivity, PPV, and predictive performance; Figure 10). During the first phase of the pandemic, around one-fifth of all cases in RNK/HD were captured in quarantine (sensitivity 19.2%; Multimedia Appendix 2), and only 1 in 20 quarantined individuals tested positive during quarantine (PPV 4.69%). Taken together, this resulted in a low predictive performance: β-weighted (2) *F*_β_-score=0.119. During phases 2 and 3 of the pandemic, sensitivity increased to 34.6% and 39.7%, and PPV increased to 14.2% and 21.2%, with *F*_β_-scores improving to 0.272 and 0.338, respectively. As contact tracing efforts were scaled back in phases 4 and 5, sensitivity decreased progressively to 9.83% and 0.693%, respectively. PPV, by contrast, continued to increase and peaked in phase 4 (25.9%) before reducing in phase 5 (19.0%). Predictive performance (*F*_β_-score weighted toward sensitivity, β=2) reduced in both the fourth (0.112) and fifth (0.009) phases.

**Figure 11:**
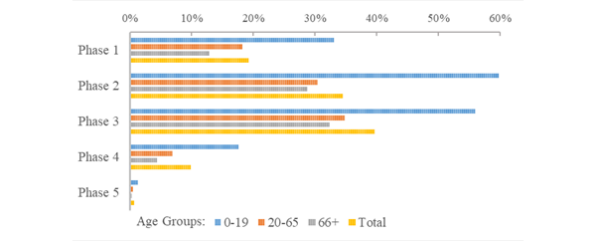
Age group quarantine sensitivity (% cases captured in quarantine) during the first 5 phases of the COVID-19 pandemic.

Comparing age groups during the period of active contact tracing (phases 1-3), quarantine sensitivity was highest in the younger population, capturing from around one-third of the infected population in quarantine in phase 1 (33.1%) to nearly two-thirds in phases 2 and 3 (59.8% and 56.0%, respectively; Figure 11; Supplementary Table S6 in Multimedia Appendix 1). Quarantine sensitivity for adults and senior citizens started at around one-tenth in phase 1 (adults 12.8%; senior citizens 8.36%), increasing to almost one-third in phase 3 (adults 34.8%; senior citizens 32.3%). Contact tracing sensitivity was lowest for senior citizens in each study phase. As contact tracing efforts were reduced in phases 4 and 5, the proportion of the infected population captured in quarantine decreased for all age groups (young: from 17.6% to 1.33%; adults: from 6.85% to 0.473%; senior citizens: from 4.36% to 0.288%).

**Figure 12:**
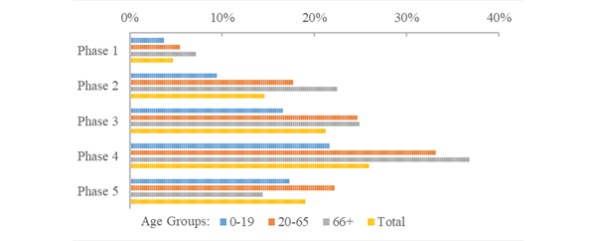
Age group quarantine PPV (% quarantine population infected) during the first 5 phases of the COVID-19 pandemic. PPV: positive predictive value.

Comparing the proportion of the quarantine population that tested positive (PPV) across different age groups, a higher proportion of adults and senior citizens in quarantine tested positive compared with younger persons in almost every study phase (Figure 12). In phase 4, when PPV was highest, 36.8% (74/201) of senior citizens placed in quarantine tested positive, 33.15% (976/2944) of adults, and 21.62% (1168/5403) of young people in quarantine tested positive (Supplementary Table S6 in Multimedia Appendix 1). In phase 3, during the period of active contact tracing, 24.9% (193/776) of senior citizens in quarantine tested positive, 24.66% (2127/8624) of adults, and 16.60% (1172/7059) of young people in quarantine tested positive.

**Figure 13:**
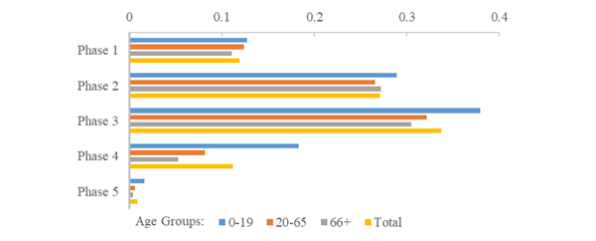
Age group quarantine predictive performance (Fβ score, weighted toward sensitivity β=2) during the first 5 phases of the COVID-19 pandemic.

The predictive performance of contact tracing (*F*_β_-weighted toward sensitivity, β=2) increased during the first 3 phases of the pandemic and decreased rapidly in the fourth and fifth phases as contact tracing efforts were reduced (*F*_β_-scores for phases 1-5=0.119, 0.272, 0.338, 0.112, and 0.009, respectively; Figure 13; Supplementary Table S6 in Multimedia Appendix 1). The predictive performance of contact tracing was consistently higher for young people compared with other age groups throughout the study phases, peaking at 0.380 in phase 3.

## Discussion

### Principal Findings

The data on SARS-CoV-2 infections and case fatalities from the RNK/HD Health Authority align with national statistics in terms of scale [3,36] and the timing of infection waves and lull periods [5]. The risks of infection, quarantine, and case fatality varied significantly across age groups. Young people exhibited the highest risks of infection and quarantine, whereas mortality and case fatalities were predominantly observed in the senior citizen population.

The overall effectiveness of contact tracing, measured by the proportion of cases captured in quarantine (sensitivity), did not reach the 80% threshold predicted to halt further COVID-19 transmission. However, the sensitivity of around 40% observed in the second and third phases of the pandemic likely contributed to reducing transmission [55,67,68]. This reduction in transmission would have helped in lowering the incidence of severe and potentially fatal infections, thus alleviating the burden on health care systems. The overall efficiency of contact tracing, measured by the proportion of quarantine cases that tested positive (PPV), improved significantly across 4 of the 5 study phases. This improvement helped to reduce the burden on noninfected individuals. The *F*_β_-score offers a consolidated measure of both the effectiveness (sensitivity) and efficiency (PPV) of contact tracing, providing a comprehensive assessment of its performance throughout the pandemic.

### Variations in Disease Outcomes

Much of the variation in COVID-19 incidence and mortality observed in RNK/HD can be attributed to the targeted release of vaccines to senior citizens and changes in the infectivity of virus variants [69,70]. Initially, when vaccine supplies were limited and prioritized for high-risk adults and senior citizens, there was a notable reduction in the risk of death among the latter group, as evidenced by decreases in both mortality and case fatality rates. At the same time, however, the increased proportion of deaths among adults suggests that the targeted vaccination efforts may not have effectively reached high-risk individuals within this age group (eg, those with preexisting conditions such as diabetes, obesity, heart failure, lung disease, or dementia). Additionally, senior citizens are known to engage more frequently in disease-preventative behaviors, both in general [71,72] and during the COVID-19 pandemic [73], which may have also contributed to better outcomes within this group.

Consistent with other published research, data from RNK/HD indicate that relatively fewer infections were reported in young people compared with adults or senior citizens during the first 2 phases of the pandemic [2,6-9]. This trend may partly be attributed to a lack of testing within this age group [10,11]. Alternatively, recent data from human challenge experiments suggest a strong correlation between symptoms and wild-type disease transmission [12]. This supports findings from other studies indicating that the initial contagion predominantly spread within the adult population where the disease was first established [9,23]. Data from the German RKI Corona-KiTa study also show that the risk of infection in young children aged 0-5 years was lower compared with other age groups during the initial phases of the pandemic [9]. An additional subanalysis of RNK/HD data confirmed that, during the early wild-type phases of the pandemic, young children aged 0-5 years had the lowest risk of infection.

### Contact Tracing and Quarantine Effectiveness and Efficiency

Data from RNK/HD show that quarantine sensitivity, PPV, and the *F*_β_-score can serve as indicators of the overall effectiveness and efficiency of contact tracing efforts. Sensitivity offers a clear measure of how effectively contact tracing captured the infected population in quarantine. To demonstrate that this success was not merely due to an increased number of people placed in quarantine, PPV (the proportion of the quarantined population who tested positive) provides a complementary measure of contact tracing efficiency. *F*_β_-scores, weighted toward sensitivity (β=2), indicate that an optimal balance between effectiveness and efficiency was achieved during the third phase of the pandemic, with the highest *F*_β_-score of 0.338, when 39.67% (3492/8803) of all infections were captured in quarantine and 21.21% (3492/16,462) of the quarantined population tested positive (Supplementary Table S6 in Multimedia Appendix 1). Unweighted *F*-scores showed similar results, but were less reliable for identifying the reduction in sensitivity between phases 3 and 4 (Supplementary Table S7 in Multimedia Appendix 1).

As a measure of overall effectiveness, quarantine sensitivity was initially low during the first study phase, likely due to the ongoing development of systems. Sensitivity increased as the risks associated with COVID-19 infection peaked, capturing nearly half of all confirmed cases during the second phase of the pandemic. As contact tracing and quarantine measures were reduced in phases 4 and 5, leading to decreased sensitivity, other infection control measures—such as widely available antigen and PCR testing—continued to help individuals identify when to self-isolate after an infection and to self-quarantine after contact with an infected person.

As a measure of overall efficiency, the PPV increased from around 5% to 27% during the first 4 phases of the pandemic. This improvement can be attributed to the increased availability of free PCR testing and the fine-tuning of other policy and operational practices. Policies exempting fully vaccinated individuals from quarantine may have also contributed to the rise in PPV. However, the PPV improved for young people despite limited vaccine availability in phases 3 and 4. Additionally, factors such as the experience and expertise of contact tracing staff and policy makers may have further enhanced the proportion of infections captured within the quarantine population.

As a combined measure of predictive performance, weighted to prioritize sensitivity over PPV, the increasing *F*_β_-scores indicate simultaneous expansion and improvement of contact tracing processes during the first 3 phases of the pandemic. Conversely, the decreasing *F*_β_-scores in phases 4 and 5 reflect the reduction in contact tracing efforts, which particularly impacted sensitivity. By incorporating both sensitivity and PPV, *F*_β_-scores offer a comprehensive measure of the overall efficiency and effectiveness of contact tracing measures.

Higher levels of quarantine sensitivity, but lower PPV and *F*_β_-scores for young people compared with adults and senior citizens, highlight the disproportionate application of quarantine measures across different age groups. One reason suggested for the higher quarantine rates among young people is their increased social interactions, such as in classroom or kindergarten settings [26]. Additionally, the conditions of quarantine for young children were often less stringent due to practical reasons, including exemptions from mask usage and the difficulty in enforcing hygiene and social distancing rules for this age group. By contrast, school closures and other restrictions were implemented to reduce such social contacts during the initial phases of the COVID-19 pandemic [74]. As schools and kindergartens reopened, additional hygiene and infection control measures were introduced to protect children from infection and minimize the need for widespread quarantine [75].

### Quarantining Young People

The decision to quarantine young people to prevent disease transmission to higher-risk groups was initially supported by contact pattern data, which suggested that young people could be a source of transmission for broader outbreaks, similar to patterns observed in influenza [25]. However, transmission data from contact tracing studies have since shown that SARS-CoV-2 transmission occurs predominantly within specific age groups and, unlike influenza, is more common in households and other settings rather than in schools [69,76-78]. This observation is supported by data from the RNK/HD area (manuscript in preparation). Additionally, data from the Corona-KiTa study indicated that infections in young adults, rather than in children or adolescents, preceded each of the early phases of infection [9].

Although the vaccine is likely to have reduced the risk of mortality and quarantine for the population of senior citizens, it seems that this protective benefit did not influence decisions regarding the quarantine of younger people. The higher levels of quarantine for younger individuals, and the apparent need to capture a greater proportion of them, were initially driven by concerns for the children themselves. These concerns included reports of unexpected pathology [16] and “long COVID” [79]. However, these complications were only anecdotally associated with children and were quickly identified as being of low incidence [14,19,22,80-84]. Relatively little attention was given to the reports of young people experiencing negative effects from quarantine [15,28,34,35,37-39,42,43], although these reports may have influenced decisions to keep schools and kindergartens open.

### Limitations

As in other locations, failures to identify cases or underreporting make it challenging to ascertain the true number of COVID-19 infections and deaths, which are likely to have been higher than reported [85]. Many cases, particularly asymptomatic and mild ones, were less likely to be tested, diagnosed, and reported. This issue was especially pertinent at the beginning of the pandemic when testing capacities were limited. Data collected for operational purposes may contain more errors and exclusions (eg, missing birth dates) due to limited validation, compared with data collected specifically for research purposes. Although case reporting in Germany was reportedly high, quarantine sensitivity, PPV, and *F*_β_-scores may still be influenced by undetected cases within the community and the quarantine population.

Measures of sensitivity, PPV, and *F*_β_-scores reported in this paper do not account for delays between testing and the notification of cases, nor the identification and notification of contact persons. Delays in case isolation and quarantine can increase the risk of disease transmission, thereby undermining the assumption that cases captured in quarantine (sensitivity) prevent all subsequent infections. To address this issue, delays would need to be quantified, along with the likelihood of resulting infections.

The size and nature of the domicile where individuals were quarantined may influence quarantine sensitivity, PPV, and *F*_β_-scores. For instance, individuals living alone may be less likely to identify or infect contact persons compared with those residing in larger families or shared living arrangements, such as aged care facilities. Institutions where staff frequently enter and exit quarantine areas and then interact with other residents may be particularly susceptible to disease transmission. Further research is needed to assess the impact of living arrangements, such as occupant density and type of domicile, on these effects.

The contribution of automated processes, such as mobile apps for detecting proximity to known cases, to the overall effectiveness or efficiency of contact tracing cannot be separately assessed from the contact tracing processes used by the RNK/HD Health Authority. A distinct evaluation of the quarantine sensitivity, PPV, and *F*_β_-score of automated or digital solutions would be valuable for comparing different contact tracing methods.

### Conclusions

During the first 3 phases of the pandemic, up to August 2021, contact tracing in the RNK/HD area played a crucial role in infection control, initially helping to limit the spread of SARS-CoV-2 and associated fatalities. During this period, high levels of quarantine sensitivity and PPV were effective in reducing virus transmission and preventing the health system from becoming overwhelmed. Especially during phases 2 and 3 of the pandemic, the availability of testing and ongoing improvements in contact tracing processes ensured that a greater proportion of the infected population was captured in quarantine (sensitivity) and that a higher percentage of those quarantined were actually infected (PPV).

The impacts of COVID-19—regarding infection, quarantine, and death—varied significantly across different age groups. Even during phases when the incidence of infection was lower in young people compared with adults, they were still significantly more likely to be placed in quarantine. Despite being up to 56 times more likely to die from an infection than adults before the introduction of the vaccine, senior citizens were significantly less likely to be placed in quarantine compared with adults or children. Urgent follow-up research is needed to clarify whether transmission predominantly occurs within specific age groups rather than between them, as has been suggested elsewhere.

In future disease outbreaks, understanding the COVID-19 pandemic and the infection control measures used will provide a valuable foundation. However, a key lesson from this pandemic is that infection control measures must be regularly adapted based on emerging information. State and federal decision makers, along with local authorities implementing policies, benefit from up-to-date data, concurrent analyses, and rapid assessments of specific risks.

This research highlights that a retrospective assessment of health authority data can offer valuable insights into past policies and their practical applications. Further analysis of contact tracing data from RNK/HD could help clarify the transmission pathways of SARS-CoV-2 within and between different age groups and social groups.

## Appendix

Multimedia Appendix 1 Data analysis.

Multimedia Appendix 2 Infection and quarantine contingency tables with quarantine sensitivity, positive predictive value, and predictive performance.

## Abbreviations

DJI: German Youth Institute
EMA: European Medicines Agency
FN: false negative
FP: false positive
GDPR: General Data Protection Regulation
IfSG: Infektionsschutzgesetz
PPV: positive predictive value
RKI: Robert Koch Institute
RNK/HD: Rhein-Neckar-Kreis/Heidelberg
RR: relative risk
RT-PCR: reverse transcription-polymerase chain reaction
STIKO: Standing Committee on Vaccination
TP: true positive
