# Supplementary material for: Contact Tracing Different Age Groups During the COVID-19 Pandemic: Retrospective Study From South-West Germany
Source: Online J Public Health Inform. 2024 Oct 29;16:e54578. doi: 10.2196/54578 (PMC11558225; doi:10.2196/54578)
Supplement: Multimedia Appendix 1 [file ojphi_v16i1e54578_app1.docx]

## Appendix - Supplementary data tables

Supplementary Table S1: Data records summary.

|  | **Age Group** | | | | |
| --- | --- | --- | --- | --- | --- |
|  | **0-19** | **20-65** | **66up** | **Unknown** | **Total** |
| **Population** | 130,387 | 437,581 | 139,006 | 0 | 706,974 |
| **All records** | 81,259 | 160,867 | 20,696 | 1,020 | 263,842 |
| **Infection/Case events** | 51,971 | 129,632 | 16,511 | 34 | 198,148 |
| **Infected individuals** | 50,056 | 125,822 | 16,263 | 34 | 192,175 |
| **Quarantine events** | 33,761 | 38,553 | 5,339 | 988 | 78,641 |
| **Quarantined individuals** | 32,092 | 36,510 | 5,220 | 988 | 74,810 |
| **Quarantined cases** | 4,473 | 7,318 | 1,154 | 2 | 12,947 |
| **Deaths** | 0 | 80 | 852 | 0 | 932 |

Supplementary Table S2. Proportion of age groups in total population, infected, quarantined and fatalities during first five phases of the COVID-19 pandemic.

Supplementary Table S3. Risks and relative risks of quarantine, infection and death during first five phases of the pandemic of young, adult and elderly people.

Supplementary Table S4. Proportion, risk and relative risk of infection, quarantine and death, in women and men, and persons living in Heidelberg and Rhein-Neckar-Kreis.

Supplementary Table S5: Risks and relative risks of quarantine, infection and death during first five phases of the pandemic of very young (0-5), young (6-19), adult (20-65) and elderly people (over 65).

Supplementary Table S6: Quarantine / contact tracing Fβ score, sensitivity, positive predictive value (PPV) and ratio of quarantine to cases within different age groups during first 5 phases of COVID-19 pandemic.

Supplementary Table S7: Infection and quarantine contingency tables with unweighted F-scores.

|  |  | Quarantined | | F-score |
| --- | --- | --- | --- | --- |
|  |  | + | - |  |
| Infected | + | TP | FN | 2TP/ (2TP+FP+FN) |
|  | - | FP | TN |  |
| Phase 1 | + | 401 | 1,686 | 0.075 |
|  | - | 8,149 | 696,738 |  |
| Phase 2 | + | 5,810 | 11,004 | 0.206 |
|  | - | 33,877 | 656,283 |  |
| Phase 3 | + | 3,492 | 5,311 | 0.276 |
|  | - | 12,970 | 685,201 |  |
| Phase 4 | + | 2,219 | 20,359 | 0.143 |
|  | - | 6,336 | 678,060 |  |
| Phase 5 | + | 1,025 | 146,841 | 0.013 |
|  | - | 4,362 | 554,746 |  |
