# Supplementary material for: Contact Tracing Different Age Groups During the COVID-19 Pandemic: Retrospective Study From South-West Germany
Source: Online J Public Health Inform. 2024 Oct 29;16:e54578. doi: 10.2196/54578 (PMC11558225; doi:10.2196/54578)
Supplement: Multimedia Appendix 2 [file ojphi_v16i1e54578_app2.pptx]

## Slide 1
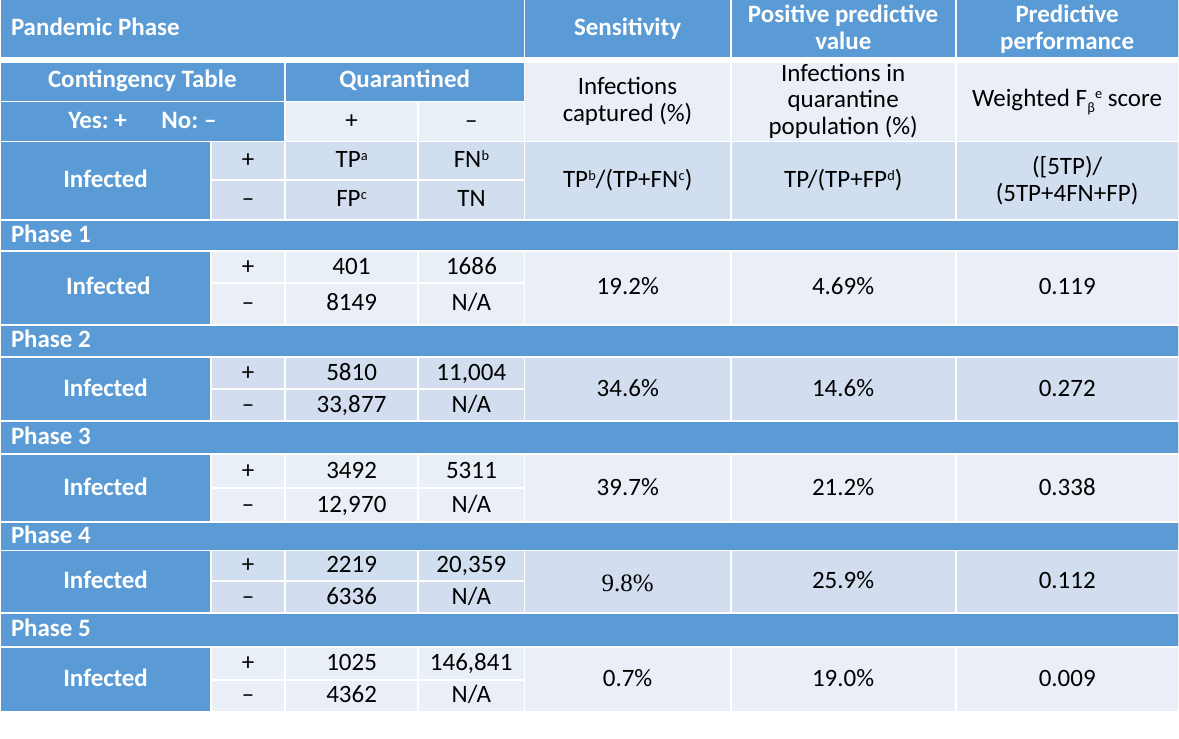

| Pandemic Phase | | | | Sensitivity | Positive predictive value | Predictive performance |
| --- | --- | --- | --- | --- | --- | --- |
| Contingency Table | | Quarantined | | Infections captured (%) | Infections in quarantine population (%) | Weighted Fβe score |
| Yes: + No: – | | + | – | | | |
| Infected | + | TPa | FNb | TPb/(TP+FNc) | TP/(TP+FPd) | ([5TP)/ (5TP+4FN+FP) |
| | – | FPc | TN | | | |
| Phase 1 | | | | | | |
| Infected | + | 401 | 1686 | 19.2% | 4.69% | 0.119 |
| | – | 8149 | N/A | | | |
| Phase 2 | | | | | | |
| Infected | + | 5810 | 11,004 | 34.6% | 14.6% | 0.272 |
| | – | 33,877 | N/A | | | |
| Phase 3 | | | | | | |
| Infected | + | 3492 | 5311 | 39.7% | 21.2% | 0.338 |
| | – | 12,970 | N/A | | | |
| Phase 4 | | | | | | |
| Infected | + | 2219 | 20,359 | 9.8% | 25.9% | 0.112 |
| | – | 6336 | N/A | | | |
| Phase 5 | | | | | | |
| Infected | + | 1025 | 146,841 | 0.7% | 19.0% | 0.009 |
| | – | 4362 | N/A | | | |
